# The mind-body dilemma: a model for understanding women's mental health

**DOI:** 10.1186/2050-2974-1-S1-O29

**Published:** 2013-11-14

**Authors:** Megan Jenkins

**Affiliations:** 1 University of Ballarat, Australia

## 

Body image dissatisfaction has been recognised as a key concern of Australian women, with longitudinal studies indicating that between 40% and 82% of women are unhappy with their body. Further, depression is the number one burden of disease among women and depressive symptoms and suicidal ideation represent a significant cost to both the individual and the community. Given the concurrently high rates of depression and body image disturbance in women, it is surprising that body image and disordered eating are not at the forefront of discussion regarding women's mental health.

The current paper will present a model of mental health for women that empirically tests and extends the Objectification Model (Frederickson & Noll). The model of mental health offers important treatment implications for recovery and early intervention in women. Based on the proposed model, it is asserted that body image should be a key focus of research if we are to adequately understand women's mental health. Further, it is proposed that body image and disordered eating should be considered as playing a role in precipitating and maintaining psychological distress in women, even when no diagnosable eating disorder is present.

This abstract was presented in the **Disordered Eating – Characteristics & Treatment** stream of the 2013 ANZAED Conference.

